# Study on the relationship between the changes in polyamine content and seedless grapes embryo rescue breeding

**DOI:** 10.3389/fpls.2024.1362989

**Published:** 2024-04-08

**Authors:** Guirong Li, Kaiwei Li, Feifei Han, Huanchao Gao, Ling Wang

**Affiliations:** ^1^ School of Horticulture and Landscape Architecture, Henan Institute of Science and Technology, Xinxiang, China; ^2^ Henan Province Engineering Research Centers of Horticultural Plant Resource Utilization and Germplasm Enhancement, Xinxiang, Henan, China

**Keywords:** seedless grape, polyamine, embryo rescue technology, *in vitro* culture, embryonic development

## Abstract

This study was envisaged to investigate the physiological reasons affecting the embryo development and abortion of seedless grapes on the basis of the previous embryo rescue breeding techniques of seedless grapes. Specifically, the relationship between the embryo rescue breeding of seedless grapes and the change of polyamine content was evaluated, in order to provide hybrid germplasm in the breeding of new seedless grape cultivars. Four ovules of 4 naturally pollinated Eurasian seedless grape cultivars, including ‘Thompson Seedless’ grape (hereinafter referred to as ‘Seedless White’ grape), ‘Flame Seedless’ grape, ‘Heshi Seedless’ grape and ‘Ruby Seedless’ grape were employed for the study. Changes in the endogenous polyamine content, exogenous polyamine content, and the suitable combination of exogenous polyamines in the seedless grape berries and isolated ovules were determined during the best embryo rescue period. Furthermore, the effect of different exogenous polyamine contents on the germination and seedling rate of different seedless grape embryos was analyzed. In the best embryo rescue period, the number of ovules had different effects on the content of polyamines. For seedless grape cultivars with 4 ovules, a high content of polyamines was found to be more beneficial in the embryonic development. The existence of embryos had different effects on the development of embryos. In the ovules with embryo, an increase in the content of polyamine was beneficial to the growth and development of the ovule. Different ratios of exogenous polyamines had varying effects on the embryonic development. Putrescine (Put) exhibited the greatest effect on the embryonic development. Further, correlation analysis showed that different combinations of exogenous polyamines had varying effects on the embryonic development. A maximal ovule development was observed in the combination of exogenous polyamines of putrescine2+spermidine2+spermine1. For maximal embryo germination and seeding formation, the optimal combination was putrescine2+spermidine2+spermine2. Irrespective to the number of ovules or the existence of embryos, the results indicated that a high content of endogenous polyamines promoted the growth and development of embryos. The embryo rescue efficiency of different exogenous polyamines was different, and the appropriate combination of exogenous polyamines was beneficial to the growth and development of ovules, with a high development rate of the ovule and seedling.

## 1 Introduction

Seedless grape breeding is an important aspect of grape research worldwide, and the proportion of seedless grapes in the current cultivation of fresh cultivars is gradually increasing. The cultivated seedless grape cultivars are mostly European grape cultivars (*Vitis vinifera* L.), which present an excellent quality, but are not disease resistant. Therefore, the breeding of seedless cultivars with high quality, large grain and stress resistance has become one of the important goals in the grape breeding research. Embryo rescue technology was first realized in 1982 (Chu et al., 2023). In recent years, the embryo rescue breeding of seedless grapes is an important technology and approach to improve the efficiency of seedless grape breeding. This technique has been widely used in seedless grape breeding (Zhu and Zhang, 2022). Although the embryo rescue technology has significantly increased the seedless rate of offspring, it still needs to be further studied and improvised in terms of the embryo development rate, germination rate and seedling rate (Li et al., 2019; Zhu et al., 2020).

The prime focus of most researchers has always been in the improvement and perfection of the embryo rescue breeding technology. Polyamines (PAs) are closely related to embryonic development. The common PAs in plants are putrescine (Put), spermidine (Spd) and spermine (Spm) (Wang et al., 2023). Many researchers have paid attention to the relationship between polyamines and embryonic development. Polyamines played an important role in the embryonic development in longan (Liang and Chen, 2005), lychee (Chen and Lv, 2000), gojiberry (Hu et al., 2001), sweet cherry (Qu et al., 2021) and rice (Guo and Tang, 1990). A low polyamine content in ovule and the sharp decrease of polyamine content during the embryonic development are the main factors leading to seed abortion (Guo et al., 2007; Hu et al., 2008; Pan et al., 2011; He et al., 2013; Zhu, 2022). Guo et al. (2007) studied the changes in the endogenous polyamines in ovules at different stages of embryo development and found that a high ratio of (Spd+Spm)/Put and Spm/Pas were beneficial to ovule development. Pan et al. (2011) studied the changes in the endogenous polyamine content in ovules at different embryonic development stages, analyzed the content of PAs and the ratio of various PA contents, and revealed the mechanism of embryo abortion of seed abortive grape. Jiao et al. (2018) studied the seedless grape using *in vitro* seedlings and evaluated different hybrid parents, sampling time and culture medium. The germination rate and plant development rate were determined, and it was found that the efficiency of the *in vitro* embryo rescue could be improved by adding appropriate amount of PAs. Zhu (2022) also studied the content and ratio of endogenous polyamines in seedless and seedless grape cultivars in different periods, and a quantitative analysis was carried out to reveal the mechanism of the embryo abortion of seed abortive seedless grape. Xu et al. (2022) studied the role of paclobutrazol in embryo rescue of fruit trees. It was found that embryos treated with different concentrations of paclobutrazol (PAC) showed different embryo germination rate and seedling rate, and the optimum PAC concentration of ovule and embryo medium was determined. The study of Wang et al. (2001) revealed that paclobutrazol could affect the gibberellin concentration, and then regulated the content of polyamines, thus regulating plant growth. This is consistent with the results of Wang (2023), wherein gibberellin led to embryo abortion by regulating the decrease of polyamine content, which indirectly indicates that polyamines are beneficial to the growth and development of plant embryos.

Polyamines are bioactive low molecular weight aliphatic nitrogen bases produced in the process of biological metabolism. PAs play an important role in the plant growth and development. PAs in plant cells mainly exist in free, covalent and non-covalent forms. There are two polyamine synthesis pathways in plants, namely arginine decarboxylation ADC pathway and ornithine decarboxylation ODC pathway. PAs binds to uronic acid or lignin, a component of cell wall, after which GA_3_ promotes Put synthesis and stimulates embryonic hypocotyl elongation. Put is known to improve the embryo development of seedless grape (Shiozaki et al., 1998; Tang et al., 2009). PAs are also known to significantly affect the *in vitro* embryo and callus culture. Exogenous PAs can increase the frequency of somatic embryogenesis. To sum up, the increase of polyamine content is the premise of somatic embryogenesis, which plays a regulatory role in the development of somatic embryos. Therefore, exploring the effects of endogenous and exogenous PAs on the abortion of immature embryos during the embryonic development of seedless grapes and obtaining a number of seedless grape embryo rescue plants can be helpful to provide hybrid germplasm in the breeding of new seedless grape cultivars. It is very important to improve the embryo rescue breeding technology of seedless grape considering the future prospects.

Most of the studies have reported the relationship between the endogenous polyamine content and embryonic development, or mostly on the ratio of endogenous polyamine content. However, there are few reports on the effects of exogenous polyamine content on the embryonic development. At the same time, the effect of sampling time on embryo rescue significantly affects the efficiency of embryo rescue, because if the sampling time is not appropriate, the embryo is difficult to save, or even aborted (Xu et al., 2022). Giancaspro et al. (2022) studied the optimal rescue time of different parent genotypic combinations. A high level of embryonic development is a necessary condition for efficient ovule formation. As a result, an optimal best sampling time plays an important role in embryo rescue (Jiao et al., 2018). Therefore, it is necessary to determine the best sampling time for each cultivar, which is a prerequisite for successful embryo rescue, and new seedless grapes are cultivated by embryo rescue technology (Li et al., 2004, 2015; Xu et al., 2022). These studies indicate that PAs are closely related to the embryonic development. Accordingly, on the basis of previous studies, this study employed the samples directly from the best embryo rescue period. In order to study the relationship between embryogenic rescue breeding of seedless grape and the change of polyamine content, the berry types with different number of ovules of the same seedless grape and the *in vitro* ovule types of different ovules of the same seedless grape were studied. Samples were collected during the best embryo rescue period, and the changes of polyamine content in different types were determined, in order to explore the effect of PAs on the embryonic development of seedless grape. In addition, the contents of endogenous polyamines and exogenous polyamines, different ratios of exogenous polyamines and their combinations in fruits and ovules of different seedless grape cultivars were determined. At the same time, in order to further study the relationship between the PAs and embryonic development, the ovules affected by different ratios of exogenous polyamines were inoculated, the development rate and seedling rate were measured, and a number of seedless grape embryo rescue seedlings were obtained. A sharp decrease of polyamine content may be one of the reasons leading to grape embryo abortion. The relationship between the polyamine content and embryonic development can provide a theoretical basis for improving the seedling rate of embryo rescue. Furthermore, the embryo rescue breeding technology of seedless grape was improved using *in vitro* culture, embryo rescue test-tube seedling rooting, seedling domestication and transplanting, and finally planted to the field. A number of hybrid offspring plants of seedless grape were obtained and provided hybrid germplasm materials for the breeding of new seedless grape cultivars. The results can lay a foundation for reducing the occurrence of grape embryo abortion.

## 2 Materials and methods

### 2.1 Test materials

**Figure 1:**
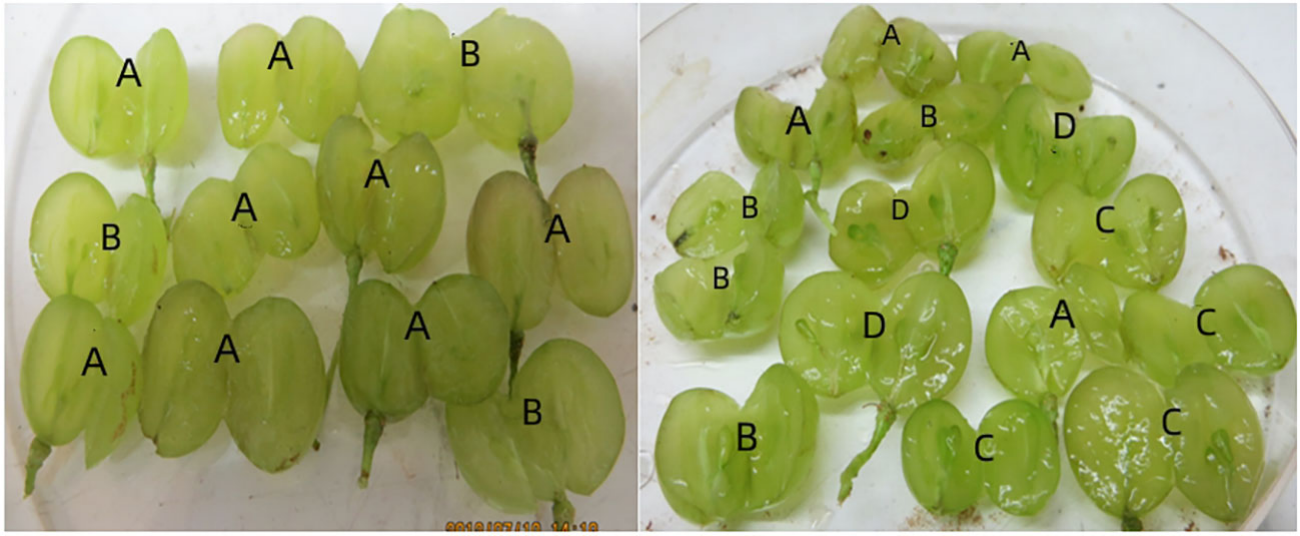
Fruit types of every seedless grape: **(A)** No ovules, **(B)** 1 ovule, **(C)** 2 ovules, **(D)** 3 ovules.

Four kinds of naturally pollinated ‘Thompson Seedless’ grape, ‘Flame Seedless’ grape, ‘Heshi Seedless’ grape and ‘Ruby Seedless’ grape were employed in the study, which were sampled and cultured during the best embryo rescue period (‘Thompson Seedless’ grape, 37d sampled; ‘Flame Seedless’ grape, 45d sampled; ‘Heshi Seedless’ grape, 60d sampled; ‘Ruby Seedless’ grape, 65d sampled). The materials were taken from the grape germplasm resource nursery of Northwest University of Agriculture and Forestry Science and Technology. Eight ears of 4 natural pollinating materials, namely ‘Thompson Seedless’ grape, ‘Flame Seedless’ grape, ‘Heshi Seedless’ grape and ‘Ruby Seedless’ grape, were randomly selected, put into a plastic foam box with ice and quickly brought back to the laboratory. The ovules were immediately stripped, counted and classified, and were divided into four types (no ovule, 1 ovule, 2 ovules and 3 ovules; **Figure 1**). 90 fruits of each type were randomly selected, and 30 fruits were divided in a single group. A total of three groups (n=3) were employed in the study. The samples were then quickly frozen with liquid nitrogen and stored in the refrigerator at -80°C, until further use.

**Figure 2:**
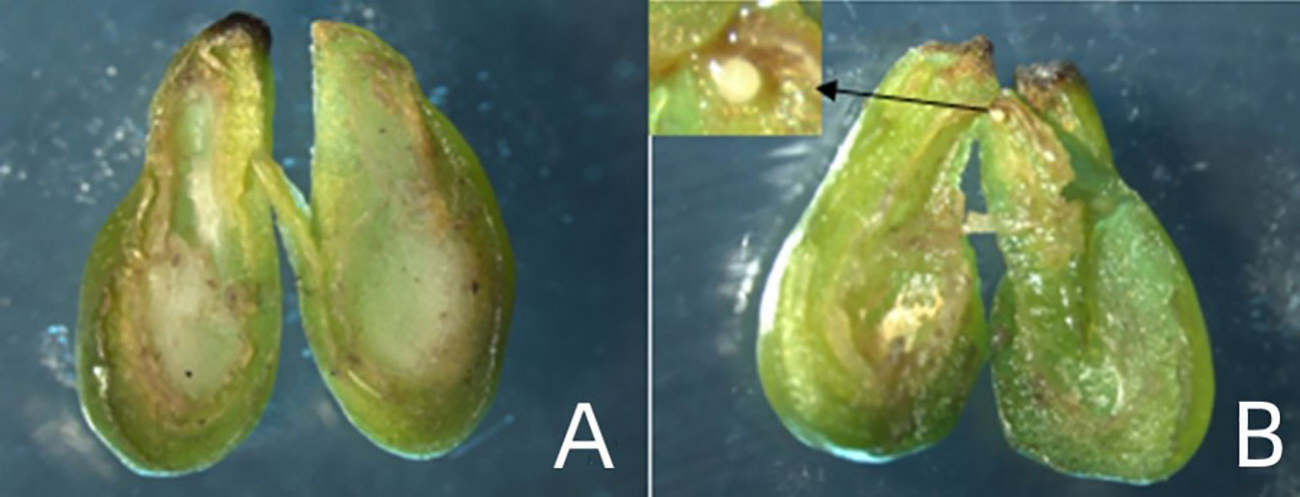
Types of *in vitro* ovules: **(A)** Absence of embryo, **(B)** Ovule containing single embryo.

After 8 weeks of *in vitro* culture, the ovules of each material were sampled and stripped, and 60 ovules with embryo and 60 ovules without embryo were selected (**Figure 2**). Each type was divided into a group of 20 ovules, a total of three groups (3 repetitions; n=3). First, the ovules were soaked in distilled water to remove the effect of the attached medium on the determination results, and then washed with deionized water for 3 times. The removed ovules and no-embryo ovules were chopped and mixed respectively, and the stripped embryos were inoculated and cultured, and then frozen in liquid nitrogen and stored in the refrigerator at -80°C.

### 2.2 Determination of endogenous free PAs in seedless grape berries

The sample of the berry preparation was extracted from the refrigerator at -80°C. According to the method of Kotzabasis et al. (1993), the endogenous free PAs were extracted and their contents were determined by high-pressure liquid chromatography (HPLC): 30 fruits were divided into one group, a total of three groups (n=3). HPLC determination was done using Shimadzu LC-10AT (Japan), the chromatographic column was a reverse C18 column (150 mm × 4.6 mm), the mobile phase was 60% methanol in water, the flow rate was 0.5ml·min^-1^, the column temperature was 25°C, and the detection wavelength of Shimadzu SPD-10A detector was 254 nm. Through the analysis of HPLC, the standard curves were plotted for Put, Spd and Spm, and the contents were quantitatively calculated by external standard method, and each type was determined repeatedly for 3 times.

### 2.3 Determination of endogenous free PAs in seedless grape ovules of *in vitro* culture

The *in vitro* culture sample prepared in advance was taken from the refrigerator at -80°C. The endogenous free PAs were extracted and their contents were determined using HPLC.

### 2.4 Effects of different ratios of exogenous PAs on the developmental status of seedless grape ovules

The seedless grape cultivars tested were naturally pollinated ‘Thompson Seedless’ grape, ‘Flame Seedless’ grape, ‘Heshi Seedless’ grape and ‘Ruby Seedless’ grape. The polyamine components (Put, Spd and Spm) were all purchased from Biorigin (Beijing) Inc. All three PAs were dissolved in water, and the experiment was formulated by an orthogonal experimental design (3 factors and 2 levels, a total of 4 treatments) (**Table 1**), which was generally prepared on the same day of the ears treatment. The pre-experiment was performed according to an earlier research Liu et al. (2003), and it was observed that the effect of spraying exogenous PAs was the best at 50 mg l^-1^. Hence, we designed the two levels of 0 mg l^-1^ and 50 mg·l^-1^. The application method was to spray the ear with a hand-held spray from 18:00 to 21:00 at night and the temperature was 22°C to 30°C, so that the whole ear was wet. One tree was used as an experimental group, and 5 ears with the same degree of development were selected for chemical spraying as a treatment, sprayed once every other week, and repeated 3 times, with absence of spraying as the control.

**Table 1:** L4(2^2^) orthogonal design table of exogenous PAs (mg·l^-1^).

Treatment^a^	Put	Spd	Spm
EP1	0	0	0
EP2	0	50	50
EP3	50	50	0
EP4	50	0	50

a Treatment of different concentrations of exogenous polyamines on seedless grape berries were divided into 4 groups: EP1, EP2, EP3, EP4.

### 2.5 Effect of different exogenous polyamine ratios on germination into seedlings formation of seedless grape embryo

Four kinds of seedless grape with natural pollination were selected (300 ovules, ovule length ≥ 2 cm, 10 ovules in 100 mL triangular flask, a total of 30 flasks). When culturing ovules *in vitro*, solid-liquid double-layer medium was used, solid medium was modified MM3 medium, and the liquid medium was ER medium. Exogenous PAs with orthogonal experimental design (3 factors, 2 levels, 4 treatments in total) were added respectively (**Table 2**). We carried out the pre-experiment with reference to the research results of Ponce et al. (2002) and Jiao et al. (2018), and found that the effect of adding exogenous PAs was the best at 2 mmol·l^-1^. Hence, the two levels for the experiment were 0 mmol·l^-1^ and 2 mmol·l^-1^. After 8 weeks of ovule culture *in vitro*, the developed embryos were inoculated onto WPM + BA 0.2 mg·l^-1^ solid medium supplemented with 500 mg·l^-1^ hydrolyzed casein, 3 g·l^-1^ activated carbon, 20 g·l^-1^ sucrose and 7 g·l^-1^ agar and pH 6.0. The embryo development rate, embryo germination rate and seedling rate were observed and counted.

**Table 2:** L4(2^2^) orthogonal design table of exogenous polyamines (mmol·l^-1^).

Treatment^a^	Put	Spd	Spm
P1	0	0	0
P2	0	2	2
P3	2	2	0
P4	2	0	2

a Treatments of different concentrations of exogenous polyamines on in vitro culture of seedless grape were divided into 4 groups: P1, P2, P3, P4.

### 2.6 Statistical analysis

For the content of endogenous free PAs, the berries and isolated ovules of each cultivar were divided into three groups (n=3). We calculated the corresponding average and standard deviation based on the results of three repetitions (n=3), and one-way analysis of variance (ANOVA) was employed using GraphPad Prism 8 (GraphPad Software Inc., San Diego, CA, USA) to analyze the data. Significant differences are showed by asterisks (* *P<0.05*, ** *P<0.01*).

## 3 Results

### 3.1 Content of endogenous free PAs in seedless grape berries

Put content: Put content in berries without ovules of ‘Thompson Seedless’ grape was 8.13 µg·g^-1^, the content in one ovule was 10.00 µg·g^-1^, the content in two ovules was 12.30 µg·g^-1^. The significance analysis showed that the content of 2 ovules was significantly higher than that of less than 2 ovules. In the berries without ovules of ‘Flame Seedless’ grape Put content was 16.32 µg·g^-1^, the content of one ovule was 19.31 µg·g^-1^, the content of two ovules was 23.02 µg·g^-1^, the content of three ovules was 26.98 µg·g^-1^. The significance analysis showed that the content of 3 ovules was significantly higher than that of less than 3 ovules. The content of 2 ovules was significantly higher than that of 1 ovule and the content of no ovule, and the content of 1 ovule was significantly higher than that of no ovule. Put content in the berries without ovules of ‘Heshi Seedless’ grape was 10.10 µg·g^-1^, the content of one ovule was 12.11 µg·g^-1^, the content of two ovules was 18.12 µg·g^-1^, the content of three ovules was 22.14 µg·g^-1^. The significance analysis showed that the content of 3 ovules was significantly higher than that of less than 3 ovules. The content of 2 ovules was significantly higher than that of 1 ovule and the content of no ovule, and the content of 1 ovule was significantly higher than that of no ovule. Put content in the berries without ovules of ‘Ruby Seedless’ grape was 18.00 µg·g^-1^, the content of one ovule was 24.03 µg·g^-1^, the content of two ovules was 28.02 µg·g^-1^, the content of three ovules was 33.21 µg·g^-1^. The significance analysis showed that the content of 3 ovules was significantly higher than that of less than 3 ovules. The content of 2 ovules was significantly higher than that of 1 ovule and the content of no ovule, and the content of 1 ovule was significantly higher than that of no ovule. The results showed that with an increasing number of ovules in these four seedless grapes, the content of Put increased, indicating that a high content of Put was beneficial for the growth and development of ovules in berries. On the contrary, when there were no ovules, the content of Put was low, that is, when the content of Put in berries was low, the growth of ovules was inhibited to a certain extent in this environment, and the number decreased until it disappeared.

**Figure 3:**
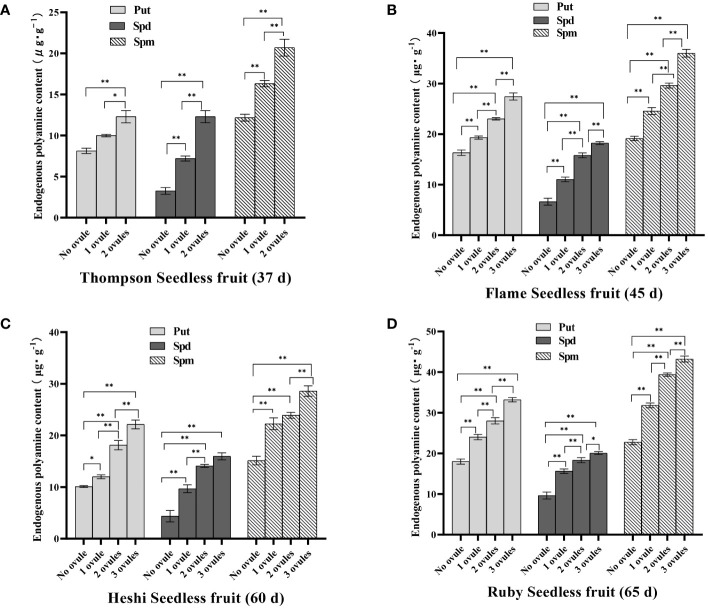
Endogenous polyamine content of different samples of 4 seedless grape fruit (the best sample time): **(A)** ‘Thompson Seedless’ grape (37 d), **(B)** ‘Flame Seedless’ grape (45 d), **(C)** ‘Heshi Seedless’ grape (60 d), **(D)** ‘Ruby Seedless’ grape (65 d). Data represented as mean ± SD, n=3. ^∗^ *p < 0.05* and ^∗∗^ *p < 0.01* indicate values that are significantly different.

Spd content: Spd content in the berries without ovules of ‘Thompson Seedless’ grape was 3.26 µg·g^-1^, the content of one ovule was 7.20 µg·g^-1^, the content of two ovules was 12.30 µg·g^-1^. The significance analysis showed that the content of 2 ovules was significantly higher than that of less than 2 ovules, and the content of 1 ovule was significantly higher than that of no ovule. Spd content in the berries without ovules of ‘Flame Seedless’ grape was 6.63 µg·g^-1^, the content of one ovule was 11.04 µg·g^-1^, the content of two ovules was 15.80 µg·g^-1^, the content of three ovules was 18.23 µg·g^-1^. The significance analysis showed that the content of 3 ovules was significantly higher than that of less than 3 ovules. The content of 2 ovules was significantly higher than that of 1 ovule and the content of no ovule, and the content of 1 ovule was significantly higher than that of no ovule. Spd content in the berries without ovules of ‘Heshi Seedless’ grape was 4.36 µg·g^-1^, the content of one ovule was 9.68 µg·g^-1^, the content of two ovules was 14.10 µg·g^-1^, the content of three ovules was 15.96 µg·g^-1^. The significance analysis showed that the content of 3 ovules was significantly higher than that of 1 ovule and the content of no ovule. The content of 2 ovules was significantly higher than that of less than 2 ovules, and the content of 1 ovule was significantly higher than that of no ovule. Spd content in the berries without ovules of ‘Ruby Seedless’ grape was 9.63 µg·g^-1^, the content of one ovule was 15.62 µg·g^-1^, the content of two ovules was 18.33 µg·g^-1^, the content of three ovules was 20.07 µg·g^-1^. The significance analysis showed that the content of 3 ovules was significantly higher than that of less than 3 ovules. The content of 2 ovules was significantly higher than that of 1 ovule and the content of no ovule, and the content of 1 ovule was significantly higher than that of no ovule. The results showed that with an increasing number of ovules in these four seedless grapes, the content of Spd increased, indicating that a high content of Spd was beneficial for the growth and development of ovules in berries. On the contrary, when there were no ovules, the content of Spd was low, that is, when the content of Spd in berries was low, the growth of ovules was inhibited to a certain extent in this environment, and the number decreased until it disappeared. It could be inferred that these three PAs were closely related to the development of seedless grape ovules, and a decrease in their content could lead to a decrease in the number of ovules, until they disappeared (**Figure 3**).

Spm content: Spm content in the berries without ovules of ‘Thompson Seedless’ grape was 12.18 µg·g^-1^, the content of one ovule was 16.32 µg·g^-1^, and the content of two ovules was 20.70 µg·g^-1^. The significance analysis showed that the content of 2 ovules was significantly higher than that of less than 2 ovules, and the content of 1 ovule was significantly higher than that of no ovule. Spm content in the berries without ovules of ‘Flame Seedless’ grape was 19.13 µg·g^-1^, the content of one ovule was 24.56 µg·g^-1^, the content of two ovules was 29.60 µg·g^-1^, the content of three ovules was 36.00 µg·g^-1^. The significance analysis showed that the content of 3 ovules was significantly higher than that of less than 3 ovules. The content of 2 ovules was significantly higher than that of 1 ovule and the content of no ovule, and the content of 1 ovule was significantly higher than that of no ovule. Spm content in the berries without ovules of ‘Heshi Seedless’ grape was 15.13 µg·g^-1^, the content of one ovule was 22.26 µg·g^-1^, the content of two ovules was 23.9 µg·g^-1^, the content of three ovules was 28.6 µg·g^-1^. The significance analysis showed that the content of 3 ovules was significantly higher than that of less than 3 ovules. The content of 2 ovules was significantly higher than that of no ovule, and the content of 1 ovule was significantly higher than that of no ovule. Spm content in the berries without ovules of ‘Ruby Seedless’ grape was 22.78 µg·g^-1^, the content of one ovule was 31.82 µg·g^-1^, the content of two ovules was 39.40 µg·g^-1^, the content of three ovules was 43.23 µg·g^-1^. The significance analysis showed that the content of 3 ovules was significantly higher than that of less than 3 ovules. The content of 2 ovules was significantly higher than that of 1 ovule and the content of no ovule, and the content of 1 ovule was significantly higher than that of no ovule. The results showed that with an increase in the number of ovules in these four seedless grapes, the Spm content increased, indicating that high Spm content was beneficial for the growth and development of ovules in berries. On the contrary, when there were no ovules, the Spm content was low, that is, when the Spm content in berries was low, the growth of ovules was inhibited to a certain extent in this environment, and the number decreased until it disappeared.

### 3.2 Content of endogenous free PAs in seedless grape ovules of *in vitro* culture

Put content: Put content in *in vitro* ovules without embryos of ‘Thompson Seedless’ grape was 0.75 µg·g^-1^, while the content of embryos was 2.32 µg·g^-1^. Put content in *in vitro* ovules without embryos of ‘Flame Seedless’ grape was 0.80 µg·g^-1^, while the content of embryos was 2.82 µg·g^-1^. Put content in *in vitro* ovules without embryos of ‘Heshi Seedless’ grape was 0.63 µg·g^-1^, while the content of embryos was 2.10 µg·g^-1^. Put content in *in vitro* ovules without embryos of ‘Ruby Seedless’ grape was 0.84 µg·g^-1^, while the content of embryos was 3.72 µg·g^-1^. Through significance analysis, the Put content of embryos was significantly higher than that of no embryo. The results indicated that these four seedless grapes selected during the optimal embryo rescue period, cultured under the same *in vitro* conditions for 8 weeks, when there were developmental embryos in the *in vitro* ovules, the content of Put in the *in vitro* ovules was higher than that in the *in vitro* ovules without embryos, indicating that the high content of Put was beneficial for the growth and development of embryos in *in vitro* ovules. On the contrary, when the content of Put was low, the growth and development of embryos were also inhibited to a certain extent in this environment and gradually abortion.

**Figure 4:**
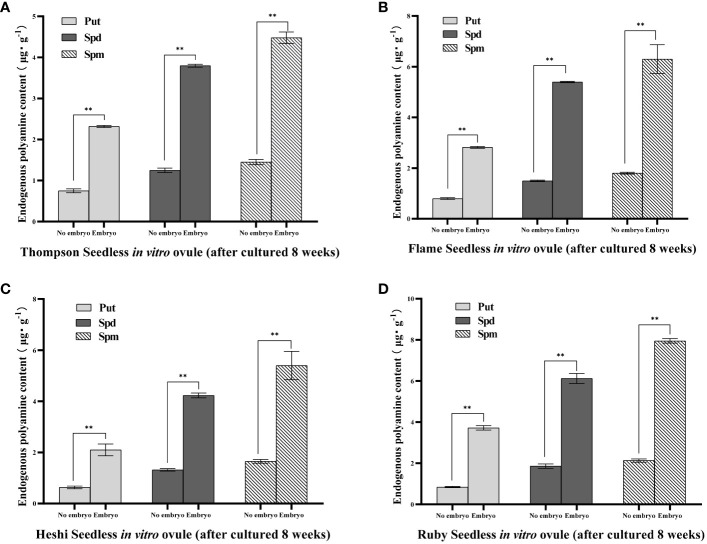
Endogenous polyamine content of different samples of 4 seedless grape *in vitro* ovule (after cultured 8 weeks): **(A)** ‘Thompson Seedless’ grape *in vitro* ovule, **(B)** ‘Flame Seedless’ grape *in vitro* ovule, **(C)** ‘Heshi Seedless’ grape *in vitro* ovule, **(D)** ‘Ruby Seedless’ grape *in vitro* ovule. Data represented as mean ± SD, n=3. ^∗∗^ *p < 0.01* indicate values that are significantly different.

Spd content: Spd content in the *in vitro* ovules without embryos of ‘Thompson Seedless’ grape was 1.25 µg·g^-1^, while the content of embryos was 3.85 µg·g^-1^. Spd content in the *in vitro* ovules without embryos of ‘Flame Seedless’ grape was 1.50 µg·g^-1^, while the content of embryos was 5.40 µg·g^-1^. Spd content in the *in vitro* ovules without embryos of ‘Heshi Seedless’ grape was 1.32 µg·g^-1^, while the content of embryos was 4.23 µg·g^-1^. Spd content in the *in vitro* ovules without embryos of ‘Ruby Seedless’ grape was 1.86 µg·g^-1^, while the content of embryos was 6.12 µg·g^-1^. Through significance analysis, the content of embryos was significantly higher than that of no embryo. The results indicated that these four seedless grapes selected during the optimal embryo rescue period, cultured under the same *in vitro* conditions for 8 weeks, when there were developmental embryos in the *in vitro* ovules, the content of Spd in the *in vitro* ovules was higher than that in the *in vitro* ovules without embryos, indicating that the high content of Spd was beneficial for the growth and development of embryos in *in vitro* ovules. On the contrary, when the content of Spd was low, the growth and development of embryos were also inhibited to a certain extent in this environment and gradually abortion. It could be inferred that the content of polyamines was closely related to the development of embryos (**Figure 4**).

Spm content: Spm content in the *in vitro* ovules without embryos of ‘Thompson Seedless’ grape was 1.45 µg·g^-1^, while the content of embryos was 4.48 µg·g^-1^. Spm content in the *in vitro* ovules without embryos of ‘Flame Seedless’ grape was 1.80 µg·g^-1^, while the content of embryos was 6.30 µg·g^-1^. Spm content in the *in vitro* ovules without embryos of ‘Heshi Seedless’ grape was 1.65 µg·g^-1^, while the content of embryos was 5.40 µg·g^-1^. Spm content in the *in vitro* ovules without embryos of ‘Ruby Seedless’ grape was 2.13 µg·g^-1^, while the content of embryos was 7.95 µg·g^-1^. Through significance analysis, the content of embryos was significantly higher than that of no embryo. The results indicated that these four seedless grapes selected during the optimal embryo rescue period, cultured under the same *in vitro* conditions for 8 weeks, when there were developmental embryos in the *in vitro* ovules, the content of Spm in the *in vitro* ovules was higher than that in the *in vitro* ovules without embryos, indicating that the high content of Spm was beneficial for the growth and development of embryos in *in vitro* ovules. On the contrary, when the content of Spm was low, the growth and development of embryos were also inhibited to a certain extent in this environment and gradually abortion.

### 3.3 Effects of different ratios of exogenous PAs on the developmental status of seedless grape ovules

(1) Analysis of the effect of different exogenous polyamine ratios on the developmental status of ovules in different seedless grape cultivars.

When 4 kinds of seedless grape berries were sprayed with different exogenous PAs ratios, among the three selected factors, the total number of ovules of each cultivar increased with an increase in the Put factor level. The total number of ovules increased with an increase in the Spd factor level. With an increase in the Spm factor level, the total number of ovules decreased. The analysis of orthogonal experiment showed that the primary and secondary effects of the three factors on the total number of ovules of the four seedless grapes were Put, Spd and Spm, which indicated that putrescine had a great effect on the ovule development of seedless grape berries, followed by spermidine and spermine, respectively.

(2) Selection of suitable exogenous polyamine content combination.

**Figure 5:**
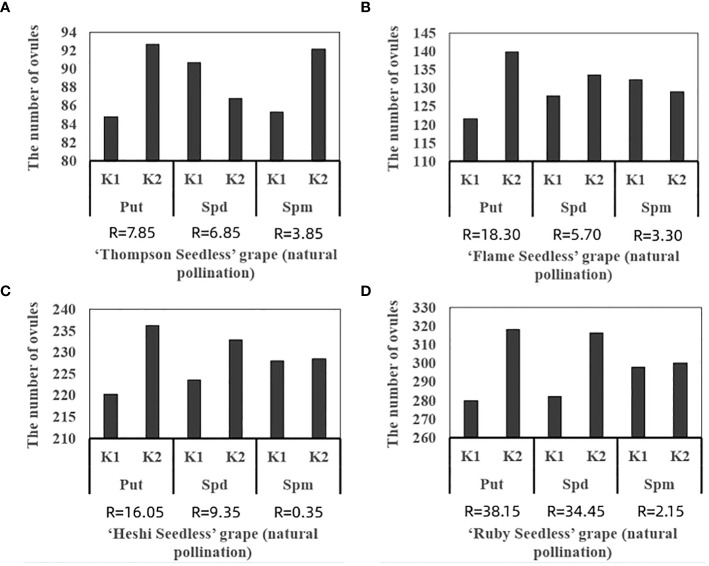
Range analysis of the total of ovules in seedless grape cultivars with different exogenous PAs: **(A)** ‘Thompson Seedless’ grape, **(B)** ‘Flame Seedless’ grape, **(C)** ‘Heshi Seedless’ grape, **(D)** ‘Ruby Seedless’ grape. K1 represents the average number of total ovules of each factor at the first level. K2 represents the average number of total ovules of each factor at the second level. R stands for range (between K1 and K2).

The average value of each factor level found that the second level of Put factor, the second level of Spd factor and the first level of Spm factor were the highest. Hence, the suitable combination of exogenous PAs content was Put2 Spd2Spm1, that is, the suitable combination of exogenous PAs content was Put 50 mmol l^-1^ + Spd 50 mmol l^-1^ (**Table 3**; **Figure 5**).

**Table 3:** Effect of different exogenous PAs on the development of seedless grape ovule.

Cultivars	Treatment^a^	No. of fruit ears	No. of fruit grains	No. of ovules
‘Thompson Seedless’ grape (natural pollination)	EP1	5	900	83.3
	EP2	5	900	86.3
	EP3	5	900	98.0
	EP4	5	900	87.3
‘Flame Seedless’ grape (natural pollination)	EP1	5	900	120.3
	EP2	5	900	122.7
	EP3	5	900	144.3
	EP4	5	900	135.3
‘Heshi Seedless’ grape (natural pollination)	EP1	5	900	215.3
	EP2	5	900	225
	EP3	5	900	240.7
	EP4	5	900	231.7
‘Ruby Seedless’ grape (natural pollination)	EP1	5	900	261.7
	EP2	5	900	298.3
	EP3	5	900	334.3
	EP4	5	900	302

a Treatments of different concentrations of exogenous PAs on seedless grape berries were divided into 4 groups: EP1, EP2, EP3, EP4.

### 3.4 Effect of different exogenous PAs on germination into seedlings formation of seedless grape embryos

(1) Analysis of the effect of different ratio of exogenous PAs on the germination and seedling rate of different seedless grape embryos.

When 4 kinds of naturally pollinated seedless grapes were rescued by embryos with different ratio of exogenous PAs, among the three selected factors, the embryo germination rate of each cultivar increased with an increase in the Put, Spd and Spm factors. The analysis of orthogonal experiment showed that the primary and secondary effects of the three factors on the total number of ovules of 4 kinds of seedless grapes were different. The primary and secondary effects of 3 factors on ‘Thompson Seedless’ grape embryo rescue were as follows: Spd, Put, Spm. The primary and secondary effects on ‘Flame Seedless’ grape embryo rescue were: Put, Spm, Spd. The primary and secondary effects on ‘Heshi Seedless’ grape embryo rescue were: Spm, Spd, Put. The primary and secondary effects on ‘Ruby Seedless’ grape embryo rescue were: Spd, Put, Spm. The results indicated that different exogenous PAs had different embryo rescue efficiency when different seedless grapes were used as female parents.

**Figure 6:**
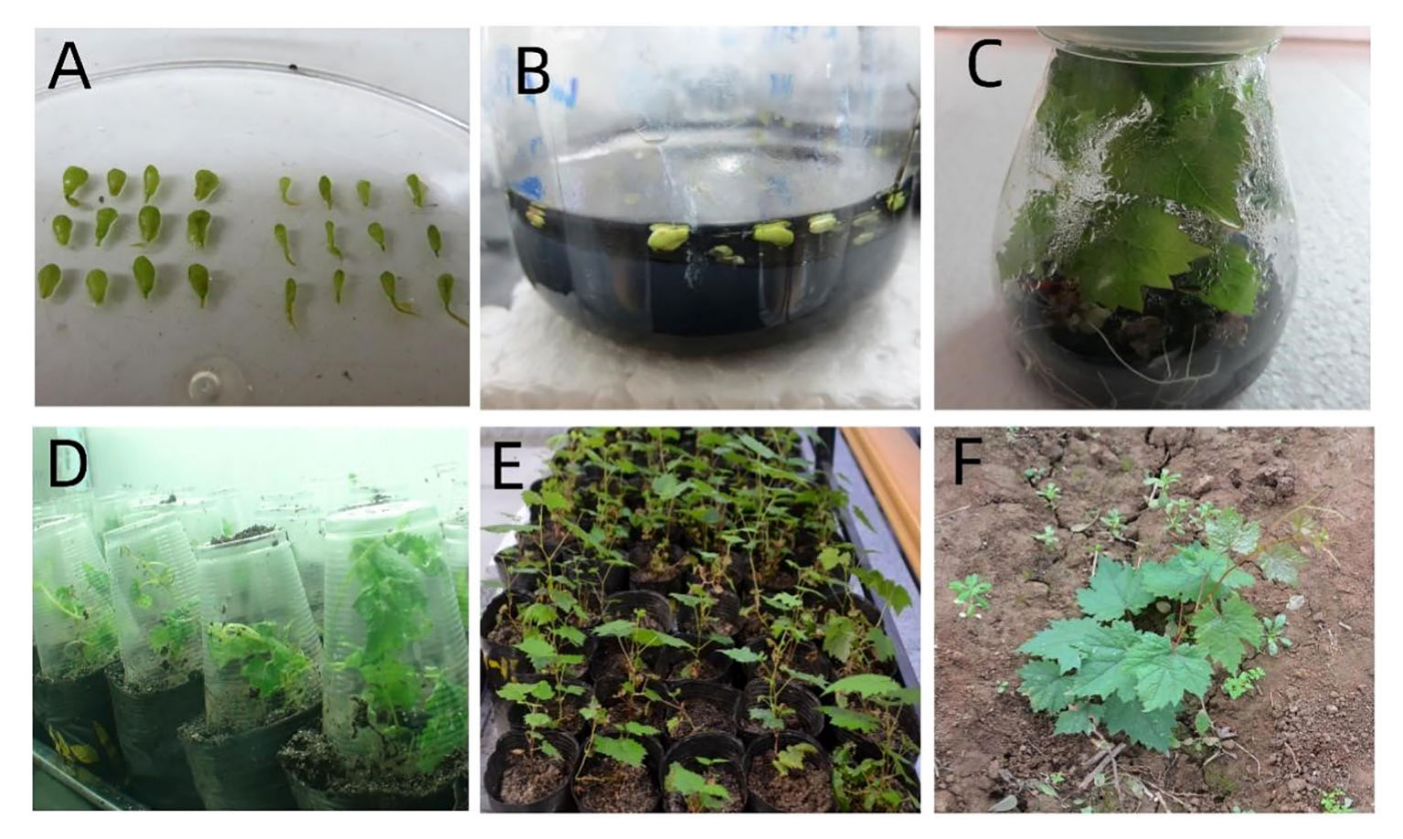
Germination and seedling formation of ovule under the best combination of exogenous polyamine concentration Put2Spd2Spm2: **(A)** *In vitro* ovules, **(B)** Ovules culture on solid-liquid medium, **(C)** Embryo germination and seedling formation, **(D)** Refining seedlings, **(E)** Domestication, and **(F)** Transplanting.

(2) Selection of suitable ratio combination of exogenous PAs:

According to the average value of each factor level, it was found that the second level of Put factor, the second level of Spd factor and the second level of Spm factor were the highest. Hence, the suitable combination of exogenous PAs was Put2 Spd2Spm2, that is, the suitable combination of exogenous PAs content was Put 2 mmol l^-1^ + Spd 2 mmol·l^-1^ + Spm 2 mmol l^-1^ (**Tables 4**, **5**; **Figure 6**).

**Table 4:** Effect of different exogenous PAs on germination into seedlings formation of seedless grape embryo.

Cultivars	Treat ment^a^	No. of ovules cultured	No. of embryos developed	% Rate of embryos developed	No. of embryos germinated	% Rate of embryos germinated	No. of plantlet developed	% Rate of plantlet developed
‘Thompson Seedless’ grape	P1	300	5	1.7	0	0.0	0	0.0
	P2	300	12	4.0	3	25.0	1	8.3
	P3	300	26	8.7	5	19.2	2	7.7
	P4	300	9	3.0	2	22.2	0	0.0
‘Flame Seedless’ grape	P1	300	29	9.7	13	44.8	3	10.3
	P2	300	36	12.0	19	52.8	8	22.2
	P3	300	49	16.3	25	51.0	11	22.4
	P4	300	35	11.7	20	57.1	11	31.4
‘Heshi Seedless’ grape	P1	300	26	8.7	11	42.3	5	19.2
	P2	300	31	10.3	17	54.8	6	19.4
	P3	300	42	14.0	27	64.3	8	19.0
	P4	300	34	11.3	15	44.1	7	20.6
‘Ruby Seedless’ grape	P1	300	48	16.0	21	43.8	5	10.4
	P2	300	59	19.7	31	52.5	14	23.7
	P3	300	65	21.7	36	55.4	29	44.6
	P4	300	57	19.0	28	49.1	11	19.3

a Treatments of different concentrations of exogenous PAs on *in vitro* culture of seedless grape were divided into 4 groups: P1, P2, P3, P4.

**Table 5:** Range analysis of germination and seedling formation rate of seedless grape embryos with different exogenous PAs.

Cultivars	Kij^a^	Put	Spd	Spm
‘Thompson Seedless’ grape	K1	4.2	0.0	3.9
	K2	3.9	8.0	4.2
	R	0.3	8.0	0.3
‘Flame Seedless’ grape	K1	16.3	20.9	16.4
	K2	26.9	22.3	26.8
	R	10.7	1.5	10.5
‘Heshi Seedless’ grape	K1	19.3	19.9	19.1
	K2	19.8	19.2	20.0
	R	0.5	0.7	0.9
‘Ruby Seedless’ grape	K1	17.1	14.9	27.5
	K2	32.0	34.2	21.5
	R	14.9	19.3	6.0

a Range analysis of germination and seedling formation rate: K1 represents the average rate of plantlet developed of each factor at the first level. K2 represents the average rate of plantlet developed of each factor at the second level. R stands for range (between K1 and K2).

## 4 Discussion

PAs are widely involved in the whole process of fruit growth and development. Biasi et al. (1988) reported that the content of free PAs in apple was consistent with the growth rate of fruit. During the first cell expansion period of the young fruit, the content of PAs was at a high level, and it was found that the peak of PAs appeared before the peak of fruit growth. Escribano and Merodio (1994) found that the content of PAs in citrus was higher in the early stages of fruit development that decreased with the growth and development of fruit, and then increased during fruit ripening. On the other hand, Chen and Lv (2000) studied that PAs were not only related to litchi embryonic development, but could be an important cause of litchi embryo abortion. Chen et al. (2006) reported that the contents of putrescine and spermidine in young fruits were higher in the early stage of fruit development in longan, and decreased with fruit development. During the period of rapid fruit expansion, the contents of Put, Spd and Spm in the aril increased rapidly and were at a high level. The polyamine content was also high in the rapid growth stage of seeds. Guo et al. (2007) determined the content of endogenous PAs in ovules during embryonic development and abortion of seedless grape ‘Jufeng’ and ‘Venus Seedless’. The results showed that the contents of the three PAs in the ovule of “Venus Seedless” decreased sharply 40 days after anthesis, and the content was much lower than that of ‘Jufeng’. This period was the key period of its embryonic development. It is suggested that the PAs were closely related to their embryonic development, and the sharp decrease of polyamine content could be one of the reasons for grape embryo abortion. The results of this experiment are consistent with the above results. In this experiment, the berry types with different number of ovules in the same seedless grape and the *in vitro* ovule types with or without different isolated ovules in the same seedless grape were studied. The changes of PAs in different types of subjects were determined. The results showed that: (1) A high content of Put in different berry types was beneficial to the growth and development of ovules in berries. When the content of Spd in berries was low, the growth of ovules was inhibited and the number of ovules decreased until they disappeared in this environment. When the content of Spm was low, the growth of ovules was inhibited and the number of ovules decreased until it disappeared. It could be concluded that these three PAs were closely related to the ovule development of seedless grape, and the decrease in their content could lead to the decrease of the number of ovules and their disappearance. (2) In the type of *in vitro* ovules with or without embryos, when there were developmental embryos in the *in vitro* ovules, the content of Put in the *in vitro* ovules was higher than that in the *in vitro* ovules without embryos, indicating that a high content of Put was beneficial to the growth and development of embryos in the *in vitro* ovules. When there were developmental embryos in the *in vitro* ovules, the content of Spd in the *in vitro* ovules was higher than that in embryos without embryos, indicating that a high content of Spd was beneficial to the growth and development of embryos in the *in vitro* ovules. On the contrary, when the content of Spd was low, the growth and development of embryos was also inhibited and aborted gradually in this environment. When there were developmental embryos in the *in vitro* ovules, the content of Spm in the *in vitro* ovules was higher than that in embryos without embryos, indicating that the high content of Spm was beneficial to the growth and development of embryos in the *in vitro* ovules. On the contrary, when the content of Spm was low, the growth and development of embryos was also inhibited and aborted gradually in this environment. In recent years, the same results were obtained in the studies on the dynamic changes of endogenous PAs during somatic embryogenesis in other plants (Shoeb et al., 2001; Vale et al., 2022).

Adding a certain amount of exogenous PAs in the process of somatic embryogenesis and development has a certain effect on the growth and development of somatic embryos. Vondráková et al. (2015) studied the effects of exogenous Put on the level and development of endogenous PAs in spruce somatic embryos. They found that the level of Put was significantly highest in the embryos, which was consistent with the results of our study. Put was the most important factor in the primary and secondary relationship among the three factors affecting the embryonic development. However, there were few reports on the effect of adding PAs on the rescue efficiency in the process of embryo rescue of seedless grape. Some researchers had only studied the effect of single exogenous polyamine factor on the embryo rescue of seedless grape (Ponce et al., 2002). Ponce et al. (2002) pointed out that Put could significantly promote the embryo development rate and seedling rate of seedless grape embryo rescue on Nistch + 2 mmol l^-1^ or 5 mmol l^-1^ in the culture of seedless cultivar ‘Perlón’. Jiao et al. (2018) found that the addition of 3 mM Put, 0.5 mM Spm or 0.3 mM Spd significantly increased the plant development or embryo germination rate in a series of combinations. These results are consistent with the results of this experiment: In this experiment, the effects of different ratio of exogenous PAs on the germination and seedling rate of different seedless grape embryos were analyzed. The results showed that different exogenous PAs exhibited varying efficiency in embryo rescue. The suitable ratio combination of exogenous PAs was Put 2 mmol l^-1^+Spd 2 mmol l^-1^+Spm 2 mmol l^-1^. Thus it could be seen that appropriate PAs can promote the growth and development of embryos. This study successfully revealed the relationship between the change of polyamine content and embryo rescue breeding of seedless grape. At the same time, this study laid a foundation for the further development of grape breeding industry and provided effective help. This study promoted the sustainable development of agriculture to a great extent. Therefore, PAs should be a noticeable factor in improving the composition of embryo rescue culture medium in the future.

## 5 Conclusion

This study shows that high levels of endogenous polyamines could promote the growth and development of embryos, no matter to the number of ovules or the existence of embryos. Different exogenous PAs exhibited varying effects on the embryo rescue, and the appropriate combination of exogenous PAs was more beneficial to the growth and development of ovules, so as to obtain higher development rate and seedling rate. By revealing the relationship between the PAs and embryonic development, this study can increase the understanding of the relationship between them, and provide a theoretical basis for improving the seedling rate of embryo rescue. Furthermore, it can be helpful to explore and dig out more ways related to PAs for improving the embryo rescue efficiency of seedless grape in the future. This study also provides hybrid germplasm materials for the breeding of new seedless grape cultivars, and the follow-up research direction can be changed to identify the related characters of the new germplasm obtained. At the same time, PAs and hormones are also closely related to embryo abortion, which can also be used as the direction of future research. This study not only deepens the role of PAs in embryo abortion, but provides a theoretical reference and technical support for the optimization of embryo rescue technical system of seedless grape in the future, and accelerates the research process of embryo rescue breeding of seedless grape. In addition, this study enables the future scholars to study and explore the relationship among PAs, hormones and embryonic development, and further improve the seedless grape embryo rescue breeding technology. Meanwhile, the focus of future research will be to study the related genes or hormone regulation of seedless grape from the point of view of molecular biology. These research directions will make a big stepping stone in the field of seedless grape embryo rescue breeding.

## Data availability statement

The original contributions presented in the study are included in the article/supplementary material. Further inquiries can be directed to the corresponding author.

## Author contributions

GL: Conceptualization, Formal analysis, Funding acquisition, Methodology, Resources, Supervision, Validation, Writing – original draft. KL: Conceptualization, Data curation, Investigation, Methodology, Software, Writing – original draft. FH: Conceptualization, Data curation, Investigation, Methodology, Writing – original draft. HG: Conceptualization, Data curation, Investigation, Methodology, Writing – original draft. LW: Conceptualization, Formal analysis, Methodology, Supervision, Writing – original draft.
